# Treatment with once‐weekly alendronate oral jelly compared with once‐weekly alendronate oral tablet for Japanese patients with primary osteoporosis: An open‐label, prospective, observational study

**DOI:** 10.1002/hsr2.107

**Published:** 2018-12-12

**Authors:** Nobukazu Okimoto, Yukari Uemura, Toru Yoshioka, Shinobu Arita, Hiroshi Tsurukami, Hajime Otomo, Satoshi Nishida, Takayuki Ogawa, Ken Hirao, Satoshi Ikeda, Hidehiro Matsumoto, Yoriko Toten, Yuji Katae, Yuichi Okazaki, Tsuyoshi Nakagawa, Akinori Sakai

**Affiliations:** ^1^ Okimoto Clinic Kure Japan; ^2^ Department of Biostatistics, Clinical Research Support Center University of Tokyo Hospital Bunkyo‐ku Tokyo Japan; ^3^ Department of Orthopaedic Surgery Shimura Hospital Hiroshima Japan; ^4^ Department of Orthopaedic Surgery Obase Hospital Miyako‐gun Fukuoka Japan; ^5^ Tsurukami Clinic of Orthopaedics and Rheumatology Tamana Japan; ^6^ Department of Orthopaedic Surgery Moji Medical Center Kitakyushu Japan; ^7^ Department of Orthopaedic Surgery Social Insurance Nogata Hospital Nogata Japan; ^8^ Department of Orthopaedic Surgery Kaisei General Hospital Sakaide Japan; ^9^ Department of Orthopaedic Surgery Hirao Clinic Hiroshima Japan; ^10^ Department of Orthopedic Surgery Ken‐Ai Memorial Hospital Onga‐gun Fukuoka Japan; ^11^ Department of Orthopaedic Surgery Sanzai Hospital Saito Japan; ^12^ Department of Orthopaedic Surgery Chugoku‐Rosai Hospital Kure Japan; ^13^ Department of Orthopaedic Surgery Akaike Kyodo Clinic Tagawa‐gun Fukuoka Japan; ^14^ Department of Orthopaedic Surgery Tobata General Hospital Kitakyushu Japan; ^15^ Department of Orthopaedic Surgery Kure‐Nakadori Hospital Kure Japan; ^16^ Department of Orthopaedic Surgery, School of Medicine University of Occupational and Environmental Health Kitakyushu Japan

**Keywords:** alendronate, bone density, drug administration routes, elderly, Japanese, osteoporosis

## Abstract

**Background and aims:**

Clinical data regarding alendronate jelly are limited. We compared the efficacy and safety of once‐weekly alendronate oral jelly with once‐weekly alendronate tablet formulations in the context of primary osteoporosis.

**Methods:**

In this 6‐month, open‐label, prospective, observational study, Japanese patients aged ≥60 years with primary osteoporosis were included from 14 primary care centres in Japan. The effects of once‐weekly alendronate oral jelly and tablet formulations on bone mineral density (BMD), bone turnover markers, and quality of life related to gastrointestinal symptoms were assessed at baseline and 6 months. Treatment was allocated by patient preference. This potentially confounding factor was adjusted for statistically.

**Results:**

In total, 170 patients were enrolled (jelly, n = 97; tablet, n = 73). Mean percent changes in radius, lumbar spine, femoral neck, and hip BMD were similar in both treatment groups at 6 months. Both formulations decreased tartrate‐resistant acid phosphatase 5b (TRACP‐5b) and procollagen 1 N‐terminal peptide (P1NP) between baseline and 6 months (by about 50% and 60%, respectively); no significant differences in mean changes were noted in these markers between groups. At 6 months, no significant differences were noted in visual analogue scale or EuroQOL five‐dimension questionnaire scores between groups. The jelly group had significantly lower scores than the tablet group in the Izumo scale domains of heartburn (−0.81, *P* = 0.0040), epigastralgia (−0.94, *P* = 0.0003), and epigastric fullness (−0.49, *P* = 0.044). During treatment, more patients discontinued for upper gastrointestinal symptoms in the tablet group (n = 4) than the jelly group (n = 1).

**Conclusions:**

Once‐weekly alendronate oral jelly 35 mg may be a suitable alternative therapeutic agent for primary osteoporosis in Japan.

## 1 INTRODUCTION

Osteoporosis is a metabolic bone disorder with skeletal fragility and deterioration of bone structure that occurs most commonly in elderly people.^1^, ^2^ The number of patients with osteoporosis, and those with fragility fractures, is rapidly increasing as a consequence of the increase in aging populations. This increasing prevalence, and the resulting fragility fractures, represents a worldwide socio‐economic burden.^3^, ^4^, ^5^, ^6^, ^7^ Aging population growth is higher in Japan than in any other country; 26% of the Japanese population is >65 years old, and the average life span is 86.99 years for Japanese women and 80.75 years for Japanese men.^8^

Bisphosphonates remain among the most frequently prescribed drugs for osteoporosis in clinical settings.^9^ Of these, alendronate has been globally used to treat patients with osteoporosis since the 1990s, reducing the risk of vertebral or hip fracture by approximately 50% compared with placebo during a 36‐month observation period in the Fracture Intervention Trial.^10^, ^11^, ^12^ Although oral bisphosphonates increase bone mineral density (BMD) by inhibiting osteoclast bone resorption, and reduce fracture risk,^13^ an exact dosage must be taken, and tablets must be swallowed with approximately 180 mL of plain water at least 30 minutes before the first meal, drink, or medication of the day. Additionally, patients cannot lie down for at least 30 minutes after taking them.

Although previous studies reported that patients taking oral bisphosphonates develop upper gastrointestinal symptoms,^14^, ^15^, ^16^ recent studies have failed to find a relationship between the use of oral bisphosphonates and upper gastrointestinal symptoms.^17^, ^18^, ^19^, ^20^ Conversely, upper gastrointestinal symptoms were not associated with the administration of oral bisphosphonates.^21^, ^22^, ^23^, ^24^, ^25^ The poor reported adherence to oral bisphosphonates is attributable to adverse drug reactions and the complexity of dosage, and around 50% of patients discontinue within a year of initiating treatment.^26^, ^27^, ^28^, ^29^, ^30^ Previous reports indicate that fracture prevention effects can only be achieved with good treatment adherence or an approximate medication possession ratio of >80%.^31^, ^32^, ^33^, ^34^, ^35^

A bisphosphonate daily oral tablet formulation was first launched in the 1990s, after which weekly and monthly oral tablet and injection formulations were sequentially developed to increase therapeutic options for patients, decrease adverse drug reactions, and improve treatment adherence.^36^, ^37^, ^38^, ^39^, ^40^, ^41^ A weekly alendronate oral jelly formulation was developed in Japan and approved and launched after a study showed its bioequivalence to the weekly alendronate tablet.^42^, ^43^ However, one of the previous bioequivalence studies involved healthy participants.^42^ Until now, the clinical therapeutic effects of oral jelly treatment have not been examined in patients with primary osteoporosis. A recent study comparing alendronate in intravenous injection and oral jelly forms found no significant differences in terms of efficacy between both formulations.^44^ However, clinical data on the effects of alendronate oral jelly are scarce.

The purposes of this study were to compare once‐weekly alendronate oral jelly and once‐weekly tablet formulations to clarify the efficacy and safety of oral jelly for Japanese patients with primary osteoporosis and to identify similarities and differences between the two formulations.

## 2 MATERIALS AND METHODS

### 2.1 Study design

This was a 6‐month, open‐label, prospective, nonrandomized, parallel‐group, observational study conducted in 14 primary care centres in Japan between August 2013 and March 2016. The study was performed in accordance with the Declaration of Helsinki. The protocol was approved by the institutional review boards of all participating centres. Prior to commencing any study procedure, the purposes and methods of this study were explained to all participants, who provided written informed consent.

### 2.2 Patients and treatment

Patients were aged ≥60 years and had primary osteoporosis according to the 2012 diagnostic criteria for primary osteoporosis of the Japanese Society for Bone and Mineral Research.^45^ Key exclusion criteria were oesophageal abnormalities such as stricture or achalasia, inability to stand or sit upright for at least 30 minutes, hypocalcaemia, hypersensitivity to bisphosphonates, secondary osteoporosis, serious cardiovascular disease, serious renal or hepatic dysfunction, and malignant neoplasm.

We administered either alendronate oral jelly 35 mg or alendronate oral tablet 35 mg once weekly to patients with osteoporosis. Patients were treated and evaluated during a 6‐month period. The treatment drugs were selected according to the patient's preference after the patient was informed of the drug characteristics. Important risk factors were collected to account for potential confounders in assessing differences between treatments. Concurrent use of antiresorptive drugs other than the treatment drugs, such as selective oestrogen receptor modulators, denosumab, or bisphosphonates, as well as bone anabolic agents like teriparatide, was prohibited.

### 2.3 Patient survey

At the time of providing written informed consent, patients concurrently answered a structured questionnaire in which they were asked about the following: if they had ever taken the incorrect medication due to confusion between similar forms, if they had ever experienced the feeling of medicine lodged in the throat, and if they had any preference for treatment with medication in either a jelly or tablet formulation. We also asked why they chose either formulation based on several categories in the questionnaire given to both groups, including “easy to swallow,” “high potential for therapeutic effect,” “new formulation,” “clear distinguishability,” “lower frequency of forgetting the medication,” “decreased frequency of adverse drug reactions,” and “easy handling.” On the basis of their responses to the above questions, we treated the patients with their preferred drug formulation.

### 2.4 Measurements of BMD and bone turnover markers

BMD was measured at the distal 1/3 radius, lumbar spine, femoral neck, or hip using dual energy X‐ray absorptiometry at baseline and 6 months at each institution. Serum samples to measure bone turnover markers were collected at each site at baseline and at 3 and 6 months. The bone turnover markers were quantified by various commercial vendors using immunoassay kits, according to the manufacturers' instructions, to detect the bone resorption marker serum tartrate‐resistant acid phosphatase 5b (TRACP‐5b; DS Pharma Biomedical Co, Ltd, Tokyo, Japan; catalogue number 22000AMX00076000) and the bone formation marker serum procollagen 1 N‐terminal peptide (P1NP; Roche Diagnostics K.K., Tokyo, Japan; catalogue number 22500AMX00891000). This allowed us to investigate both bone resorption and formation from the same samples.

### 2.5 Assessment of QOL by the Izumo scale questionnaire, visual analogue scale (VAS), and EuroQOL five‐dimension questionnaire (EQ‐5D)

The Izumo scale is a useful disease‐specific tool that comprehensively evaluates patients' quality of life (QOL) based on gastrointestinal symptoms; it has been validated among Japanese patients.^46^ The questionnaire consists of five domains and 15 questions. In the present study, three domains (heartburn, epigastralgia, and epigastric fullness) and nine questions were used to assess QOL derived from upper gastrointestinal symptoms at baseline and at 1, 3, and 6 months. The EQ‐5D^47^ was used to measure preference‐based and health‐related QOL from baseline to 3 and 6 months. The VAS^48^ was used to assess lower back pain from baseline to 3 and 6 months.

### 2.6 Treatment persistence and adverse events (AEs)

After each QOL assessment (at baseline and 1, 3, and 6 months), we asked patients additional questions to assess treatment persistence, including whether they wished to change the alendronate formulation being administered. If the answer was affirmative, we asked them to provide reasons. We collected and aggregated all AEs, regardless of whether they had a causal relationship with the treatment drugs.

### 2.7 Statistical analysis

All obtained data were analysed using SAS software, version 9.4 (SAS Institute, Cary, North Carolina) by one author (Y.U.). All statistical tests were two‐sided, and statistical significance was set at *P* ≤ 0.05.

We aggregated the number of patients per reply (yes, occasionally yes, or no) for each question and calculated the proportion of patients. After testing for normality, baseline characteristics between groups were compared using Student's *t* test for continuous variables and the chi‐square test for categorical variables. Comparisons of percent changes from baseline to each visit point in BMD, changes in BMD, VAS scores, EQ‐5D scores, and Izumo scale scores between groups were also performed using Student's *t* test. Comparisons of percent changes from baseline to each visit point in bone turnover markers were compared using the Wilcoxon rank‐sum test.

Finally, to calculate the mean values of percent change in BMD, the BMD values for each individual at baseline and 6 months after treatment were used. Additionally, to compare mean QOL scores over time between groups, we applied a mixed‐model approach for repeated measures using post‐treatment QOL scores at each visit as response values. As the treatment drugs were allocated according to patient preference, we adjusted potential confounding factors by including them in the model as explanatory variables. For explanatory variables, in addition to group and visit (time), age, history of fracture, use of gastrointestinal drugs, use of nonsteroidal anti‐inflammatory drugs (NSAIDs), and baseline QOL scores were included to account for confounding between groups. Additionally, patients were included as a random effect in order to consider the correlation among patients. The coefficient for each group represents the mean QOL difference between the treatment drugs over the study period.

## 3 RESULTS

### 3.1 Patient disposition and drug formulation preference

**Figure 1:**
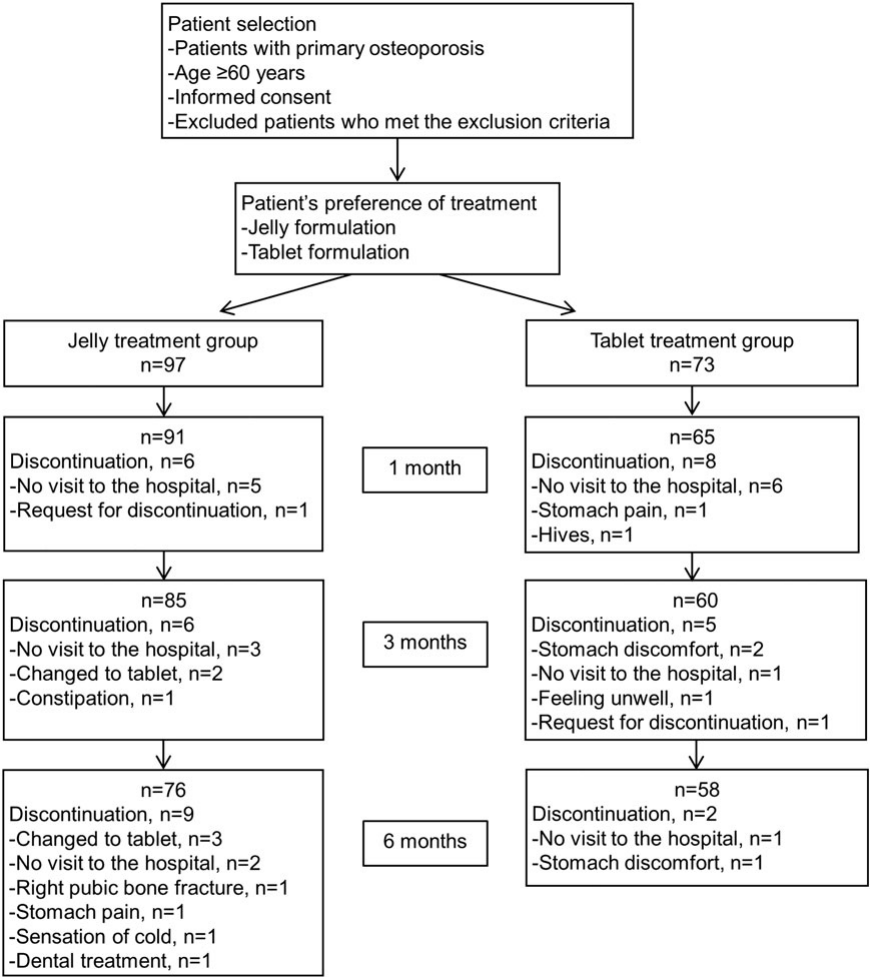
Study design and patient disposition

A total of 170 patients (jelly treatment group, n = 97; tablet treatment group, n = 73) were enrolled in the present study, from 14 institutions. Details of patient disposition at 1, 3, and 6 months per treatment group are shown in Figure 1.

Table 1 shows the answers of patients based on their experiences while taking medication in general and reasons for their preference of drug formulation at baseline. In the jelly treatment group, 21.6% of patients responded “yes” or “occasionally yes” when asked whether they had experienced taking the wrong medicine, and 24.8% of patients experienced the sensation of having medicine lodged in the throat. In the tablet treatment group, 6.9% of patients answered “yes” or “occasionally yes” to both questions. Additionally, we asked patients why they preferred the jelly or tablet formulation. The most common answer was “easy to swallow,” and the proportion was 52.6% and 83.6% in the jelly and tablet treatment groups, respectively. Regarding the reasons for patient preference for the alendronate jelly formulation, 10.3% of patients responded that they preferred it for its perceived high potential for therapeutic effects, 13.4% for its new formulation, 32.0% for its clear distinguishability, 16.5% because they forgot their medication less often, and 8.2% because of a perceived lower risk of adverse drug reactions.

**Table 1:** Patient experience while taking medication and reasons for preference of drug formulation at baseline

		Jelly	Tablet
		n (%)	n (%)
Experience of taking the wrong medication	Yes	1 (1.0)	1 (1.4)
	Occasionally yes	20 (20.6)	4 (5.5)
	No	76 (78.4)	68 (93.2)
Experience of feeling medication lodging in the throat	Yes	6 (6.2)	1 (1.4)
	Occasionally yes	18 (18.6)	4 (5.5)
	No	73 (75.3)	68 (93.2)
Reasons for preference for jelly or tablet formulation	Easy to swallow	51 (52.6)	61 (83.6)
	High potential for therapeutic effects	10 (10.3)	2 (2.7)
	New formulation	13 (13.4)	1 (1.4)
	Clear distinguishability	31 (32.0)	1 (1.4)
	Decrease in forgetting to take medicine	16 (16.5)	1 (1.4)
	Decrease of adverse drug reaction	8 (8.2)	0
	Easy handling	7 (7.2)	8 (11.0)
	Others	4 (4.1)	6 (8.2)

### 3.2 Baseline characteristics

Table 2 shows baseline characteristics of patients in both treatment groups. Patients in the jelly and tablet treatment groups had a mean ± standard deviation (SD) age of 76.47 ± 8.00 years and 75.48 ± 6.46 years, respectively. In both groups, most patients were female (94.8% and 93.2%, respectively). The proportions of patients with a history of any fracture (spine, hip joint, coccyx, wrist joint, or others) in the jelly and tablet treatment groups were 74.2% and 64.4%, respectively. Regarding drugs acting on the gastrointestinal tract, a total of 60.8% of patients in the jelly treatment group and 60.3% in the tablet treatment groups were using proton pump inhibitors, H2 blockers, or mucosal protectants. Further, 28.9% and 32.9% of patients in the jelly and tablet treatment groups, respectively, were using NSAIDs.

**Table 2:** Baseline characteristics of patients by treatment group

Characteristic		Jelly (n = 97)		Tablet (n = 73)		*P* Value^a^
		n	% or Mean ± SD	n	% or Mean ± SD	
Sex, n (%)	Female	92	94.8	68	93.2	0.62
	Male	5	5.2	5	6.8	‐
Age, years, mean ± SD		97	76.47 ± 8.00	73	75.48 ± 6.46	0.39
Menopause, years, mean ± SD		16	48.94 ± 3.07	23	49.57 ± 1.73	0.42
Body mass index, kg/m^2^, mean ± SD		95	22.45 ± 3.70	73	22.45 ± 3.15	1.00
Radius, % YAM, mean ± SD		41	66.95 ± 11.30	7	70.57 ± 14.09	0.45
Lumbar, % YAM, mean ± SD		55	71.69 ± 11.38	66	73.83 ± 15.08	0.39
Femoral neck, % YAM, mean ± SD		47	62.43 ± 8.01	60	65.33 ± 11.01	0.13
Hip, % YAM, mean ± SD		29	67.76 ± 7.92	30	73.3 ± 11.48	0.04
TRACP5b, mU/dL, median (IQR)		97	463.0 (379.0, 611.0)	61	494.0 (394.0, 666.0)	0.15
P1NP, ng/mL, median (IQR)		93	54.00 (40.50, 71.70)	60	57.85 (39.95, 74.20)	0.77
VAS, mm, mean ± SD		96	41.46 ± 27.59	73	37.20 ± 25.68	0.31
EQ‐5D, mean ± SD		96	0.67 ± 0.15	73	0.70 ± 0.16	0.27
History of all fractures, n (%)		72	74.2%	47	64.4%	0.17
History of vertebral fractures, n (%)		60	61.9%	38	52.1%	0.21
Number of vertebral fractures, n (%)		60	81%	38	54%	0.24
Use of proton pump inhibitor, n (%)		16	16.5%	10	13.7%	0.67
Use of H2 blocker, n (%)		9	9.3%	10	13.7%	0.27
Use of mucosal protectant, n (%)		37	38.1%	31	42.5%	0.32
Total use of gastrointestinal drugs %, n (%)		59	60.8%	44	60.3%	0.94
Use of NSAIDs, n (%)		28	28.9%	24	32.9%	0.33
Use of vitamin D3, n (%)		51	52.6%	40	54.8%	0.47

Abbreviations: EQ‐5D, EuroQOL five‐dimension (questionnaire); IQR, interquartile range; NSAID, nonsteroidal anti‐inflammatory drug; P1NP, serum procollagen 1 N‐terminal peptide; SD, standard deviation; TRACP‐5b, serum tartrate‐resistant acid phosphatase 5b; VAS, visual analogue scale; YAM, young adult mean.

a Student's *t* test for continuous variables and the chi‐square test for categorical variables.

There were no significant differences in baseline factors between groups, except for hip BMD. The young adult mean (YAM) hip BMD in the jelly treatment group was significantly lower than that in the tablet treatment group (67.76 ± 7.92% vs 73.3 ± 11.48%, *P* = 0.04, *t* test).

### 3.3 Changes in BMD after 6 months of treatment

Table 3 shows the percent change in BMD in the radius, lumbar spine, hip, and femoral neck from baseline to 6 months.

**Table 3:** Percent changes in BMD from baseline to 6 months

	Jelly	Tablet	*P* Value (Jelly vs Tablet)^a^
Radius			0.29
n	31	6	
% change, mean ± SD	1.1 ± 2.7	−0.1 ± 0.7	
*P* value vs baseline^a^	0.028	0.79	
Lumbar spine			0.31
n	44	49	
% change, mean ± SD	4.6 ± 7.2	3.3 ± 4.7	
*P* value vs baseline^a^	0.0001	<0.0001	
Femoral neck			0.37
n	39	47	
% change, mean ± SD	1.5 ± 7.7	2.3 ± 4.9	
*P* value vs baseline^a^	0.40	0.0028	
Hip			0.70
n	22	18	
% change, mean ± SD	1.7 ± 3.2	2.2 ± 5.3	
*P* value vs baseline^a^	0.022	0.09	

Abbreviations: BMD, bone mineral density; SD, standard deviation.

a *t* test.

**Figure 2:**
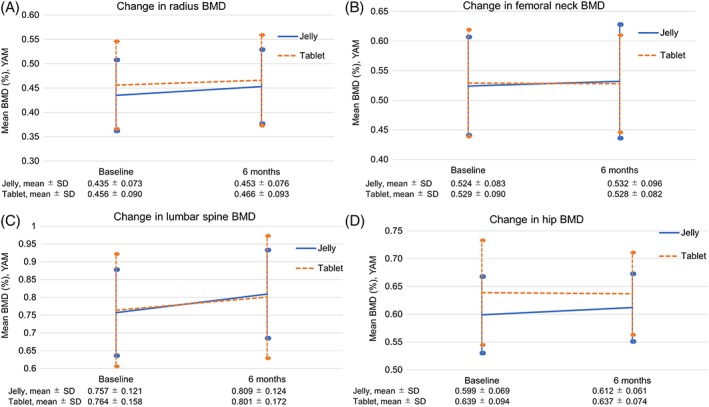
Time course changes in mean values of BMD in the, A, radius, B, femoral neck, C, lumbar spine, and, D, hip from baseline to 6 months. Abbreviations: BMD, bone mineral density; YAM, young adult mean; SD, standard deviation

Figure 2 shows the time course changes in mean BMD (% YAM) in the radius, femoral neck, lumbar spine, and hip from baseline to 6 months. Changes in BMD in all sites were not significantly different between the two treatment groups.

### 3.4 Changes in bone turnover markers after 3 and 6 months of treatment

**Figure 3:**
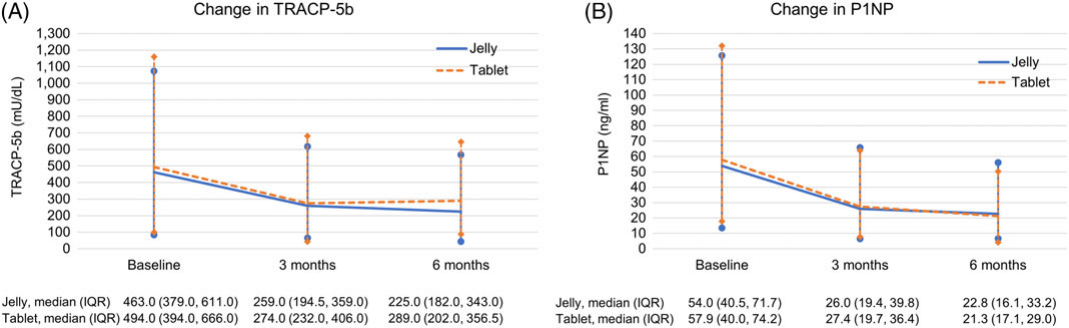
Time course changes in median (IQR) values of, A, TRACP‐5b and, B, P1NP from baseline to 3 and 6 months. Abbreviations: TRACP‐5b, serum tartrate‐resistant acid phosphatase 5b; P1NP, serum procollagen 1 N‐terminal peptide; IQR, interquartile range

Baseline values of TRACP‐5b and P1NP are shown in Table 2, and time course changes in median (interquartile [IQR]) values of those from baseline to 3 or 6 months, in Figure 3. Median (IQR) percent changes in TRACP‐5b decreased significantly (*P* < 0.0001) in the jelly and tablet treatment groups, respectively, by 42% (22%, 56%) and 40% (24%, 51%) at 3 months and 47% (33%, 66%) and 50% (26%, 57%) at 6 months. Median (IQR) percent changes in P1NP decreased significantly (*P* < 0.0001) in the jelly and tablet treatment groups, respectively, by 46% (33%, 61%) and 48% (39%, 60%) at 3 months and 57% (38%, 69%) and 57% (46%, 69%) at 6 months, respectively. No significant differences were noted between treatment groups in the mean values of either TRACP‐5b (*P* = 0.15, *P* = 0.24, and *P* = 0.12, Wilcoxon rank‐sum test) or P1NP (*P* = 0.77, *P* = 0.86, and *P* = 0.75, Wilcoxon rank‐sum test) at baseline and 3 and 6 months, respectively.

### 3.5 Changes in QOL and VAS

**Figure 4:**
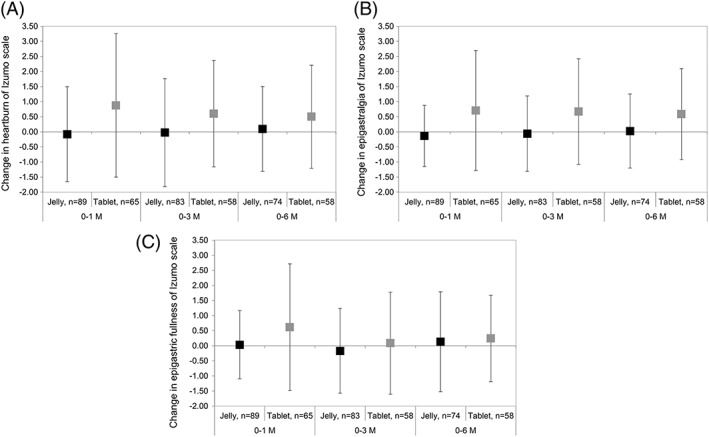
Change in Izumo scale score domains: A, heartburn, B, epigastralgia, and, C, epigastric fullness from baseline to 1, 3, and 6 months

Figure 4 shows changes in the Izumo scale scores from baseline to 1, 3, and 6 months. The mean (±SD) change in the heartburn domain was −0.08 (±1.58) and 0.88 (±2.38) at 1 month, −0.02 (±1.79) and 0.60 (±1.77) at 3 months, and 0.09 (±1.41) and 0.50 (±1.71) at 6 months in the jelly and tablet treatment groups, respectively. The mean (±SD) change in the epigastralgia domain was −0.13 (±1.01) and 0.71 (±1.99) at 1 month, −0.06 (±1.25) and 0.67 (±1.75) at 3 months, and 0.03 (±1.23) and 0.59 (±1.51) at 6 months in the jelly and tablet treatment groups, respectively. The mean (±SD) change in the epigastric fullness domain was 0.03 (±1.13) and 0.62 (±2.10) at 1 month, −0.17 (±1.40) and 0.09 (±1.69) at 3 months, and 0.14 (±1.66) and 0.24 (±1.43) at 6 months, respectively. Significant differences between the two groups were found in the heartburn domain at 1, 3, and 6 months (*P* = 0.0032, 0.041, and 0.14, respectively, *t* test) and in the epigastralgia domain at 1, 3, and 6 months (*P* = 0.0008, 0.0044, and 0.021, respectively). No significant differences were observed in the epigastric fullness domain at 1, 3, and 6 months (*P* = 0.029, 0.33, and 0.70, respectively, *t* test).

Changes in the VAS and EQ‐5D scores are shown in Table 4. No significant differences were noted between the two treatment groups in VAS and EQ‐5D scores. VAS scores showed significant lower back pain reduction at 3 and 6 months in each group. In both the jelly and tablet treatment groups, VAS scores were significantly different at 0 vs 3 months (n = 82, *P* = 0.002; n = 58, *P* = 0.0008, respectively, paired *t* test) and 0 vs 6 months (n = 73, *P* = 0.0197; n = 58, *P* = 0.0002, respectively, paired *t* test). EQ‐5D scores showed significant improvements in health‐related QOL at 3 and 6 months in both groups; however, there were no differences between the groups. In both the jelly and tablet treatment groups, EQ‐5D scores were significantly different at 0 vs 3 months (n = 82, *P* = 0.0041; n = 58, *P* < 0.0001, paired *t* test) and 0 vs 6 months (n = 73, *P* = 0.0039; n = 58, *P* = 0.0356, paired *t* test).

**Table 4:** Change in VAS scores and EQ‐5D scores from baseline to 6 months

		0‐1 mo				0‐3 mo				0‐6 mo			
		n	Mean ± SD	*P* Value^a^	Between‐group *P* Value^a^	n	Mean ± SD	*P* Value^a^	Between‐group *P* Value^a^	n	Mean ± SD	*P* Value^a^	Between‐group *P* Value^a^
VAS	Jelly	88	−3.85 ± 18.70	0.0564	0.82	82	−7.61 ± 21.26	0.002	0.69	73	−8.86 ± 30.35	0.0197	0.67
	Tablet	64	−3.17 ± 17.91	0.1619		58	−9.01 ± 19.31	0.0008		58	−10.80 ± 20.43	0.0002	
EQ‐5D	Jelly	–	–	–	–	82	0.04 ± 0.12	0.0041	0.21	73	0.06 ± 0.10	0.0039	0.74
	Tablet	–	–	–	–	58	0.05 ± 0.14	<0.0001		58	0.04 ± 0.14	0.0356	

Abbreviations: EQ‐5D, EuroQOL five‐dimension (questionnaire); SD, standard deviation; VAS, visual analogue scale.

a *t* test.

Table 5 shows a comparison of the mean Izumo scale scores over time between groups, adjusting for confounding factors using a mixed‐model, repeated‐measures approach. The jelly treatment group had significantly lower scores than the tablet treatment group in all Izumo scale domains: −0.81 (95% CI, −1.35 to −0.26; *P* = 0.0040) for heartburn, −0.94 (95% CI, −1.44 to −0.44; *P* = 0.0003) for epigastralgia, and −0.49 (95% CI, −0.97 to −0.013; *P* = 0.044) for epigastric fullness, using linear mixed‐effects models. No significant differences in Izumo domain scores were observed for other factors, including visit, age, history of vertebral fracture, use of gastrointestinal drugs, and use of NSAIDs.

**Table 5:** Between‐group comparison of the averaged Izumo scale scores over time adjusting for baseline factors using a linear mixed‐effect model, repeated‐measures approach

Variable		Heartburn			Epigastralgia			Epigastric Fullness		
		Coefficient Value	95% CI	*P* Value	Coefficient value	95% CI	*P* Value	Coefficient Value	95% CI	*P* Value
Intercept		2.45	(−0.37, 5.26)	0.093	1.33	(−1.14, 3.80)	0.287	1.67	(−0.78, 4.13)	0.179
Baseline score		0.66	(0.52, 0.81)	<0.0001	0.87	(0.697, 1.042)	<0.0001	0.70	(0.573, 0.823)	<0.0001
Treatment	Tablet	Reference			Reference			Reference		
	Jelly	−0.81	(−1.35, −0.26)	0.0040	−0.942	(−1.44, −0.44)	0.0003	−0.49	(−0.97, −0.013)	0.044
Visit	1 month	Reference			Reference			Reference		
	3 months	0.007	(−0.23, 0.24)	0.95	0.13	(−0.05, 0.32)	0.16	−0.16	(−0.35, 0.04)	0.12
	6 months	0.12	(−0.13, 0.36)	0.34	0.24	(0.05, 0.43)	0.014	0.090	(−0.12, 0.29)	0.41
Age	1 year	−0.028	(−0.07, 0.01)	0.15	−0.018	(−0.051, 0.016)	0.30	−0.018	(−0.051, 0.015)	0.28
History of vertebral fracture	No	Reference			Reference			Reference		
	Yes	0.39	(−0.18, 0.97)	0.18	0.09	(−0.41, 0.59)	0.72	0.036	(−0.46, 0.54)	0.89
Use of gastrointestinal drug	No	Reference			Reference			Reference		
	Yes	−0.064	(−0.74, 0.61)	0.85	0.29	(−0.57, 0.59)	0.98	0.134	(−0.45, 0.71)	0.65
Use of NSAIDs	No	Reference			Reference			Reference		
	Yes	−0.15	(−0.78, 0.48)	0.64	0.28	(−0.86, 0.26)	0.29	−0.05	(−0.61, 0.50)	0.85

Abbreviations: CI, confidence interval; NSAID, nonsteroidal anti‐inflammatory drug.

### 3.6 Treatment persistence and AEs

In terms of treatment persistence, when patients in the jelly treatment group were asked (at baseline and 1, 3, and 6 months) if they wished to switch to alendronate in tablet formulation, five patients (from baseline to 6 months) chose to switch to the tablet formulation. The reason given was that they experienced difficulty when swallowing the jelly.

AEs that led to discontinuation are shown in Figure 1. In the first month, the most common reason for discontinuation in both groups (jelly treatment group, n = 5; tablet treatment group, n = 6) was “did not attend hospital visit.” In the third month, the main reasons for discontinuation were “did not attend hospital visit” (n = 3) in the jelly treatment group and “stomach discomfort” (n = 2) in the tablet treatment group. At 6 months, the most common reason for discontinuation in the jelly treatment group was “switched to tablet” (n = 3). In the tablet group, two patients discontinued at this time: one for “stomach discomfort” and one for “did not attend hospital visit.” Notably, from baseline to 6 months, more patients discontinued for upper gastrointestinal symptoms (ie, “stomach discomfort” or “pain”) in the tablet treatment group (n = 4) than did patients in the jelly treatment group (n = 1).

In the present study, a total of four AEs (right pubic bone fracture [n = 1], stomach pain [n = 1], sensation of cold [n = 1], and constipation [n = 1]) occurred in the jelly treatment group. Eight AEs (rib bone fracture [n = 1], L4 fracture [n = 1], stomach pain [n = 1], stomach discomfort [n = 3], feeling unwell [n = 1], and hives [n = 1]) occurred in the tablet treatment group.

AEs possibly related to the study drug were three in the jelly treatment group and six in the tablet treatment group. Those possibly related to the study drug in the jelly treatment group were stomach pain (n = 1), sensation of cold (n = 1), and constipation (n = 1). In the jelly treatment group, the patient with constipation discontinued at 3 months, and the other two patients (one with stomach pain and the other with sensation of cold) discontinued at 6 months (Figure 1). The six AEs related to the study drug in the tablet treatment group were stomach pain (n = 1), stomach discomfort (n = 3), feeling unwell (n = 1), and hives (n = 1). Of these, the patient with stomach pain and the other with hives discontinued the study at 1 month; two patients with stomach discomfort and one who felt unwell discontinued at 3 months; and one patient with stomach discomfort discontinued at 6 months (Figure 1). Three patients presented with fractures. One in the jelly treatment group presented with a right pubic bone fracture and discontinued the study drug. In the tablet treatment group, two patients experienced fractures: one a rib bone fracture and the other an L4 fracture. Both patients continued the tablet treatment. These fractures were not considered causally related to either of the study drugs or formulations.

## 4 DISCUSSION

The main purpose of the present study was to verify the efficacy and safety of once‐weekly alendronate oral jelly 35 mg and to clarify similarities and differences compared with once‐weekly alendronate 35 mg tablet for patients with primary osteoporosis in routine clinical practice. Alendronate oral jelly 35 mg was developed in Japan to prevent gastrointestinal symptoms and reduce the choking hazard associated with the tablet formulation and was shown to be bioequivalent to the alendronate 35‐mg tablet.^42^, ^43^ There are no apparent differences in terms of efficacy between the formulations.

In the present study, when patients were asked to select a preferred treatment, some patients preferred the jelly formulation over the tablet formulation. The perceived reasons for this were “high potential for therapeutic effects,” “new formulation,” “clear distinguishability,” “forgot medication less often,” or “fewer adverse drug reactions,” but not “easy to swallow” according to the questionnaire. From these findings, we infer that patients may hesitate when choosing a new drug because they occasionally forgot to take their medicine and experienced adverse drug reactions when using previous drug treatments.

In the present study, at baseline, the hip BMD was significantly lower in the jelly group versus the tablet group, and the BMD at all other sites tended to be lower in the jelly group versus the tablet group. The percentage increase in both the hip and femoral neck BMD from baseline to 6 months was lower in the jelly group versus the tablet group (hip, 1.7% vs 2.2%; femoral neck, 1.5% vs 2.3%). The between‐group differences were not statistically significant. However, the percentage increase in the radius and lumbar spine BMD was higher in the jelly group versus the tablet group (radius, 1.1% vs 0.1%; lumbar spine, 4.6% vs 3.3%) and was increased significantly in the jelly group, although the between‐group differences were not significant. We presume that the differences observed between hip + femoral neck and radius + lumbar spine were a result of the relatively small sample size and may not be related to the effectiveness of the jelly or tablet. Further study is needed to evaluate the differences in effectiveness between the two formulations. Kunisaki et al recently performed a comparative study of sodium alendronate in intravenous injection and oral jelly form in gastric cancer patients.^44^ They found that BMD increased in both groups, and there were no significant differences between groups in terms of efficacy.

Additionally, we assessed the effects of the once‐weekly treatment with the alendronate jelly and tablet formulations on bone turnover markers. Although the time course changes in the mean values of bone turnover markers (TRACP‐5b and P1NP) did not differ significantly between the two treatment groups in this study, both treatment formulations significantly decreased the level of TRACP‐5b by approximately 50% and that of P1NP by approximately 60% from baseline to 6 months (both *P* < 0.0001). Similarly, in the study by Kunisaki et al,^44^ both treatments decreased TRACP‐5b and P1NP over time. On the basis of these results, we consider that the jelly formulation has an equivalent potential to achieve similar therapeutic effects compared with the tablet formulation. Further, these first results in patients with primary osteoporosis support the findings of the bioequivalence study performed in healthy subjects.^42^

Regarding the QOL outcomes, although EQ‐5D and VAS scores significantly improved in time with both treatment formulations, changes in EQ‐5D and VAS scores were not significantly different between the two groups. However, there were notable changes in Izumo scale scores between the two groups. The scores of all domains in the jelly treatment group remained almost unchanged at all assessment time points; however, in the tablet treatment group, Izumo scale scores increased sharply at 1 month and gradually decreased at 3 and 6 months. Additionally, in the jelly treatment group, significantly more favourable results were observed throughout the study compared with those in the tablet treatment group in the heartburn and epigastralgia domains of the Izumo scores.

In the study by Yoshioka et al,^49^ comparing oral minodronate with oral alendronate tablets, scores in the alendronate group were significantly elevated at some time points, starting at 2 weeks of treatment administration. However, in the minodronate group, none of the scores for heartburn, epigastralgia, and epigastric fullness differed significantly from baseline during the treatment period, which is similar to the present findings in the jelly treatment group. De Groen et al reported that patients taking oral alendronate developed upper gastrointestinal disorders such as oesophagitis,^16^ and it was recommended that bisphosphonate should be taken with ≥180 mL of water.^1^, ^16^ The differences in changes in Izumo scale scores in this study may imply that the jelly caused less stimulation of the upper gastrointestinal tract mucosa compared with the tablet. Thus, our findings suggest good tolerability of the novel jelly formulation among patients with primary osteoporosis.

We expected that patients taking the jelly formulation would experience greater ease in swallowing the jelly, which was the case for most patients in that group. However, five patients experienced difficulties when swallowing the jelly and switched to the tablet formulation during the treatment period, while no patients in the tablet group switched to the jelly formulation. In Japan, tablets and capsules are the most popular presentations, and most patients do not have experience with taking medication in a jelly formulation. It is possible that patients were not accustomed to taking the jelly formulation; thus, this may have been why they switched formulations. Originally, a similar jelly formulation was developed for administration to patients with dysphagia, to enable easier swallowing of the medication.^50^ With a similar intent, Okabe et al developed a film formulation that turns into jelly when the film absorbs saliva or water in the mouth. In their study comparing the oesophagus passage time of the film formulation drug and a gelatin capsule in healthy subjects,^51^ they found that the passage time of the film formulation was shorter than that of the gelatin capsule.^51^ In our study, it is likely that the passage time of the jelly through the oesophagus was shorter compared with the tablet.

This was a non‐randomized study, and patients were given the opportunity to select their preferred formulation; thus, our study closely reflects the real clinical setting in Japan. Further, the five patients who chose to switch to tablet formulation continued their treatment up to the end of the study period. Nonetheless, this study has several limitations. First, patients were allocated to the jelly or tablet treatment groups according to their preference. Although both groups were well balanced in terms of baseline characteristics (except for hip BMD), and despite adjusting for potential confounding factors using the mixed model, the study may be subject to bias because we did not quantify serum 25‐hydroxyvitamin D levels, and we did not assess family history of fracture, smoking, or other relevant factors. Second, the sample size was relatively small, which may have led to a lack of statistical power in some outcomes, such as percent changes in BMD in the radius, hip, femoral neck, and lumbar spine from baseline to 6 months; time course changes in the mean BMD in all sites; time course changes in the mean levels of TRACP‐5b and P1NP; some Izumo domain scores; and VAS and EQ‐5D scores. Third, there is a two‐fold difference in the cost of the jelly compared with that of the tablet. After applying the elderly health care subsidy, the actual difference in cost (jelly‐tablet [brand version]) for patients is 200 JPY/month, which might be considered a small difference in cost by patients, considering the advantages of the drug. However, we consider that this factor might have been a source of bias at the time our patients chose the medication to be used during this study. Further, this difference in cost may have been another reason why five patients in the jelly group switched to the tablet group. Fourth, the answer options in the questionnaire were fixed and may have influenced the patients' responses, which may not reflect their actual experiences. Fifth, the Izumo scale consists of three domains for upper gastrointestinal tract evaluation (heartburn, epigastralgia, and epigastric fullness) and two domains for lower gastrointestinal tract evaluation (constipation and diarrhoea). As bisphosphonate use is specifically associated with upper gastrointestinal tract symptoms, we only included the domains for upper gastrointestinal tract symptoms. Finally, the study was a short‐term trial. We observed patients under treatment for only 6 months. In general, intervention trials of osteoporosis should be designed to follow up patients for longer than 12 months,^10^, ^11^, ^12^, ^41^ although short‐term trials on minodronic acid have been conducted.^49^, ^52^ As the observation period of the present study was shorter than usual, further studies with longer observation periods (> 12 months) are needed to validate our results.

In conclusion, in this comparative study between the once‐weekly oral alendronate jelly and tablet formulations, we found that the therapeutic effects in BMD and bone turnover markers were almost equivalent; however, the amount of change in Izumo scale scores was significantly different between the two treatment groups. Though our results cannot readily be generalized in view of the study limitations, the findings comprise the first clinical evidence of the efficacy and safety of once‐weekly alendronate oral jelly 35 mg for the treatment of patients with primary osteoporosis in routine clinical practice. Alendronate oral jelly may be a suitable alternative therapeutic agent to treat osteoporosis.

## FUNDING

Medical writing services were funded by Teijin Pharma Ltd. The authors confirm that the sponsor was not involved in the design of the study, the enrolment of patients, or the collection, analysis, or interpretation of the data.

## CONFLICTS OF INTEREST

N. Okimoto has received consulting fees from Asahi‐kasei Pharmaceutical Co, Ltd, and payment for lectures, including speakers' bureau fees, from Asahi‐kasei Pharmaceutical Co, Ltd, Astellas Pharma Inc, Chugai Pharmaceutical Co, Daiichi‐Sankyo Co Ltd, Eisai Co, Ltd, Mitsubishi‐Tanabe Pharma Corp, Ono Pharmaceutical Co, Pfizer Japan Inc, Shionogi & Co, Ltd, Takeda Pharmaceutical Co, and Teijin Pharma Ltd. Y. Uemura has received consulting fees from Teijin Pharma Ltd. H. Tsurukami has received payment for lectures, including speakers' bureau fees, from Asahi‐kasei Pharmaceutical Co, Ltd, Astellas Pharma Inc, Chugai Pharmaceutical Co, Daiichi‐Sankyo Co, Ltd, Eli Lilly Japan K.K., Janssen Pharmaceutical K.K., Mitsubishi‐Tanabe Pharma Corp, Ono Pharmaceutical Co, Sanwa Kagaku Kenkyusyo Co, Ltd, and Teijin Pharma Ltd. S. Ikeda has received payment for lectures, including speakers' bureau fees, from Chugai Pharmaceutical Co, Daiichi‐Sankyo Co, Ltd, Pfizer Japan Inc, Takeda Pharmaceutical Co, and Teijin Pharma Ltd. A. Sakai has received grants from Astellas Pharma Inc, Asahi‐kasei Pharmaceutical Co, Ltd, Eisai Co, Ltd, Daiichi‐Sankyo Co, Ltd, Teijin Pharma Ltd, Chugai Pharmaceutical Co, Ltd, and Merck Sharp & Dohme (MSD); consulting fees or honoraria from Asahi‐kasei Pharmaceutical Co, Ltd; and payment for lectures, including speakers' bureau fees, from Asahi‐kasei Pharmaceutical Co, Ltd, Eisai Co, Ltd, Taisho Toyama Pharmaceutical Co, Ltd, and Chugai Pharmaceutical Co, Ltd. T. Yoshioka, S. Arita, H. Otomo, S. Nishida, T. Ogawa, K. Hirao, H. Matsumoto, Y. Toten, Y. Katae, Y. Okazaki, and T. Nakagawa declare that they have no conflicts of interest.

We, the authors, confirm that none of the conflicts of interest reported here had any effect on the design, conduct, or reporting of the study.

## AUTHOR CONTRIBUTIONS

Conceptualization: Nobukazu Okimoto, Akinori Sakai

Formal analysis: Yukari Uemura

Funding acquisition: Nobukazu Okimoto

Investigation: Nobukazu Okimoto, Toru Yoshioka, Shinobu Arita, Hiroshi Tsurukami, Hajime Otomo, Satoshi Nishida, Takayuki Ogawa, Ken Hirao, Satoshi Ikeda, Hidehiro Matsumoto, Yoriko Toten, Yuji Katae, Yuichi Okazaki, Tsuyoshi Nakagawa

Supervision: Akinori Sakai

Writing – original draft preparation: Nobukazu Okimoto, Yukari Uemura

Writing – review and editing: Nobukazu Okimoto, Yukari Uemura
